# An Improved Scalable Hydrogel Dish for Spheroid Culture

**DOI:** 10.3390/life11060517

**Published:** 2021-06-03

**Authors:** Jonard Corpuz Valdoz, Dallin J. Jacobs, Collin G. Cribbs, Benjamin C. Johnson, Brandon M. Hemeyer, Ethan L. Dodson, Jordan A. Saunooke, Nicholas A. Franks, Peter Daniel Poulson, Seth R. Garfield, Connor J. Knight, Pam M. Van Ry

**Affiliations:** Department of Chemistry and Biochemistry, Brigham Young University, Provo, UT 84602, USA; jcvaldoz@byu.edu (J.C.V.); djjacobs@byu.edu (D.J.J.); ccribbs@byu.edu (C.G.C.); benj821@byu.edu (B.C.J.); hemeyer2@byu.edu (B.M.H.); ethanld@byu.edu (E.L.D.); saunookj@byu.edu (J.A.S.); naf425@byu.edu (N.A.F.); ppoulson@byu.edu (P.D.P.); sethrg@byu.edu (S.R.G.); cjk96@byu.edu (C.J.K.)

**Keywords:** 3D culture, hydrogel, spheroid, primary-derived spheroid, ultra-low adherence, scaffold-free, micropatterned dish, agarose

## Abstract

Research in fields studying cellular response to surface tension and mechanical forces necessitate cell culture tools with tunability of substrate stiffness. We created a scalable hydrogel dish design to facilitate scaffold-free formation of multiple spheroids in a single dish. Our novel design features inner and outer walls, allowing efficient media changes and downstream experiments. The design is easily scalable, accommodating varying numbers of microwells per plate. We report that non-adherent hydrogel stiffness affects spheroid morphology and compaction. We found that spheroid morphology and viability in our hydrogel dishes were comparable to commercially available Aggrewell™800 plates, with improved tunability of surface stiffness and imaging area. Device function was demonstrated with a migration assay using two investigational inhibitors against EMT. We successfully maintained primary-derived spheroids from murine and porcine lungs in the hydrogel dish. These features increase the ability to produce highly consistent cell aggregates for biological research.

## 1. Introduction

Three-dimensional (3D) cell culture models are sought after for their ability to replicate in vivo tissue characteristics [1]. 3D cell culture models are based around cell aggregates or spheroids that exhibit advanced cell-to-cell interactions similar to those found in in vivo environments, making them useful for tissue engineering, regenerative medicine, and disease modeling applications [2,3,4]. Early 3D culture techniques utilized the hanging drop method, where cells are placed in droplets and inverted on a cell plate lid [5,6]. Because of variability in the time of formation using this method and difficulty in its application, anti-adherent 3D culture systems were developed. These commonly utilize scaffold-free techniques, which force cells to aggregate and form spheroids without the need for extracellular matrix proteins or other scaffolds [7]. Typically, scaffold-free culture involves the use of ultra-low adherence (ULA) flasks or plates that force cells to aggregate. ULA surfaces have previously been achieved with the use of culture plastic-reactive coatings like poly-HEMA [8] and biocompatible hydrogels including agarose [9] and agar [10]. Although previous methods using ULA have successfully developed biological models, they are either low-output with between 6 and 96 ULA well format or micropatterned, allowing for greater output but non-scalable. Zanoni et al. have shown that these non-micropatterned ULA methods produce cell aggregates of variable size [11]. Moreover, media changes in ULA and micropatterned plates frequently result in loss of spheroids as they are aspirated with the media [5]. This is exacerbated by multiple rounds of media changes necessary for complete media change when using either conventional plate formats due to limited efficiency [12].

To address these problems, we developed scalable micropatterned ULA culture dishes by casting agarose hydrogel. Our novel design features inner and outer walls that create space to assist in media changing and cell seeding. These features reduce the risk of accidental removal of aggregates in addition to minimizing the inefficiencies of multiple media changes that are necessary for many ULA plates, such as 96-well ULA plates, and the micropatterned Aggrewell™800. Our scalable design allows for customized dishes to accommodate a variable number of cell aggregates which is an improvement on current ULA and micropatterned formats. Due to the scalable nature of our dishes, researchers can produce customized dishes with specific hydrogel stiffness to fit their research needs. This overcomes inconsistent aggregate size as seen in some fixed ULA formats. Our data show the effects of differing ULA hydrogel stiffness in the formation of consistently sized and shaped aggregates. The variety of applications when using our scalable hydrogel dish is demonstrated by the generation of tumor spheroids for a migration assay and culture of primary-derived spheroids from porcine and murine lungs.

## 2. Materials and Methods

### 2.1. 3D Printing and Silicone Casting

Two molds were used in this paper—a 3D-printed positive mold and a silicone negative mold. The positive mold was designed and modeled with SolidWorks CAD and 3D printed using Formlabs Form2 with methacrylated photopolymer clear resin (Formlabs, Somerville, MA, USA). The negative mold was created by casting the Silicone Mold Making kit (Aeromarine, San Diego, CA, USA) in the 3D-printed positive mold as described by the manufacturer. The silicone negative mold was then removed from the positive mold, sterilized using 70% ethanol for at least 30 min, and dried at 45 °C. Before experiments, the silicone cast was UV irradiated inside a level 2 biosafety cabinet for at least 30 min. The silicone casts are reusable and stored in tissue culture (TC) dish at room temperature to ensure the stability of the material.

### 2.2. Hydrogel Casting

Molecular biology grade agarose powder (Fisher Scientific, Hampton, NH, USA) was dissolved in the corresponding volume of DMEM/F12 50:50 mix (Difco, Detroit, MI, USA) depending on the desired concentration and then boiled using a standard microwave oven until dissolved. Inside a biosafety cabinet, boiled agarose solution was poured over the silicone mold and left to solidify for 10 to 15 min at room temperature. Once solidified, hydrogel plates were transferred into appropriate TC dishes. The hydrogel dishes were then UV irradiated for at least 30 min before use. For storage, approximately 3 mL of basic culture media (i.e., serum-free DMEM/F12) was added to each TC dish, and each dish was sealed with Parafilm to maintain hydrogel hydration. The hydrogel plates were stored in a 4 °C fridge for up to a maximum of 2 weeks.

### 2.3. Young’s Moduli Derivation

The Young’s moduli (*E*) of the agarose, hydrogel dishes were approximated using destructive compression tests on various concentrations of agarose. The 35 mm × 9.1 mm discs of 0.5%, 1%, 2%, 3%, and 4% agarose in phosphate-buffered saline (PBS) were cast. Each disc was crushed using a Mini Instron, and resistant forces and respective displacement were measured. The modulus for each disc was calculated as described by the manufacturer using the equation, (*E* = *ε*/*σ*), where ε is the stress, and σ is the strain. The approximate Young’s modulus of each agarose hydrogel was plotted in GraphPad Prism.

### 2.4. Media Change Simulation

To assess the percentage of original media remaining after a media change in our agarose plate, 5 mL of 0.01 g/mL of fluorescein isothiocyanate (FITC, Sigma-Aldrich, St Louis, MO, USA) simulating original media was transferred to the culture chamber of an agarose plate. During the media change, the dish was tilted so that media flowed over the inner wall and into the channel where samples of the solution were collected; the rest of the solution was removed through aspiration. This process was repeated twice and twelve 100 µL samples were collected from each round. Most commercially manufactured ULA plates suggest using a 50% media refreshment three times (see PerkinElmer^®^ CellCarrier™ 3D cell culture user guide, Corning^®^ spheroid microplate protocols, and STEMCELL™ Technologies Aggrewell™800 datasheet). To replicate this suggested media change, the initial fluorescein isothiocyanate solution was placed in a 96-well plate (200 µL per well), representing the original media. The “medium” was changed by the suggested 50% media refreshment three times. Twelve 100 µL samples of each dilution were collected, and FITC concentrations of each media change were measured using a Biotek Synergy H4 plate reader. The excitation for FITC was set at 485/20, and the emission filter at 528/50. Relative fluorescence intensities were then normalized to the PBS (vehicle) fluorescence (0%) and the initial solution fluorescence (100%). This experiment was repeated three times by independent researchers.

### 2.5. Media Formulations

The complete growth medium was composed of DMEM/F12 50:50 (Difco, Detroit, MI, USA) media supplemented with 10% FBS (VWR Seradigm, Radnor, PA, USA) and 1X Penicillin-Streptomycin-Amphotericin antibiotic-antimycotic solution (Caisson Labs, Smithfield, UT, USA). The 3D growth medium was composed of complete growth medium supplemented with 0.024% methylcellulose [13]. The primary cell growth medium was composed of complete growth medium supplemented with endothelial cell growth supplement (FITC, Sigma-Aldrich, St Louis, MO, USA), and 0.024% methylcellulose was used in the 3D primary cell growth media.

### 2.6. Cell and Spheroid Culture

**Figure 1:**
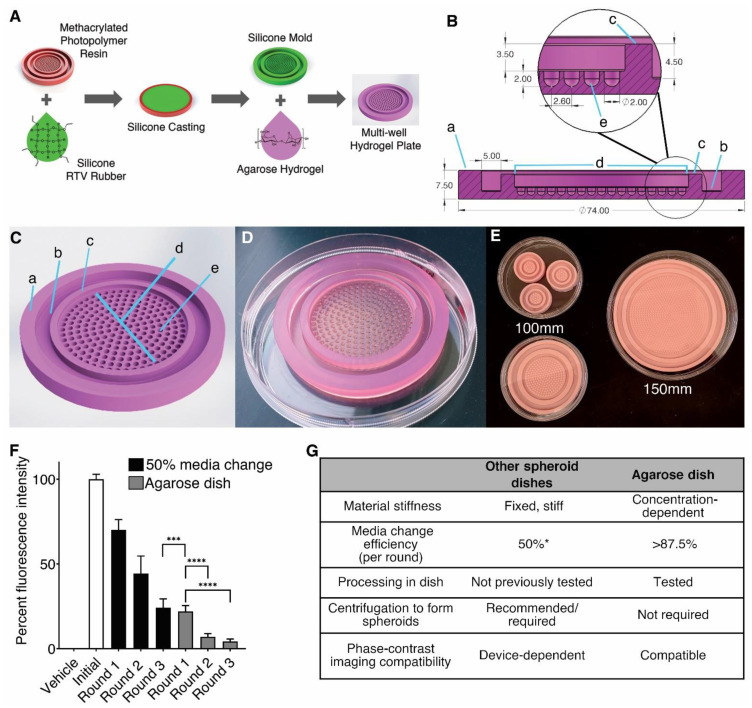
Creation of a scalable, micropatterned hydrogel mold. (**A**) Workflow for creating a multi-well casted hydrogel plate for 3D culture. Methacrylated photopolymer resin is 3D printed to create a cast for the room-temperature vulcanizing (RTV) silicone negative mold. The silicone negative mold is used to create the final multi-well hydrogel plate for 3D culture. (**B**) Agarose hydrogel dish cross-section (a = outer wall, b = channel, c = inner wall, d = culture chamber, and e = culture pocket or recess). Dimensions expressed in mm. (**C**) Agarose hydrogel dish CAD render. (**D**) Photo of a molded 217-well micropatterned agarose hydrogel dish in a standard 100 mm tissue culture dish. The dish was cast using 2% agarose in DMEM/F12 media. (**E**) Silicone rubber molds in 3 scaled sizes: (top left) 19-well, (bottom left) 217-well, and (right) 919-well. Left molds are in a 100 mm tissue culture dish while the right mold is in a 150 mm dish for reference. (**F**) Media change simulation using fluorescein isothiocyanate (FITC) in PBS. Fifty percent media change was used to simulate media change in commercially available dishes. Data normalized to vehicle control (0%) and initial solution (100%). Bars indicate the means and error bars indicate the SD of 36 technical replicates from 3 independently run experiments by independent researchers. *** *p* < 0.001; **** *p* < 0.0001, one-way ANOVA with Welch’s correction. (**G**) Table comparing the major advantages of the agarose dish over commercially available micropatterned plates. * suggested media change in most commercially available spheroid plates.

Three cell lines were used in this study: wild-type human lung epithelial carcinoma, A549 (ATCC^®^ CCL-185™), endothelial EAhy (EA.hy.926, ATCC^®^ CRL-2922™) lentivirally transduced with Azurite gene, and lung fibroblast HFL1 (ATCC^®^ CCL-153™) transduced with mCherry gene. These cells were maintained in complete growth medium. Cells were dissociated using 0.25% Trypsin-EDTA (Gibco, Waltham, MA, USA) and counted using a Bio-Rad automated cell counter. Three milliliters of 3D growth media containing the appropriate number of cells was seeded into the culture chamber (Figure 1(Bd,Cd)). The specific cell seeding densities used are found in the specific sections below. The agarose dish cultures were not spun down by centrifugation to form spheroids. The cultures were incubated at 37 °C with 5% CO2. The medium was changed every two to three days. Images were taken using a phase-contrast microscope (Amscope, Irvine, CA, USA).

For spheroid culture in Aggrewell™800 24-well plates, Aggrewell™ plates were pretreated as suggested by the manufacturer using Anti-adherence rinsing solution (STEM cell Technologies). Cells were seeded by adding one milliliter of 3D growth media with suspended cells into each well. The cell count per spheroid was kept similar between the agarose and the Aggrewell™ plates. The plates were then centrifuged at 300× *g* for 3 min. After which, the 3D cultures were incubated at 37 °C in a 5% CO2 humidified incubator. The medium was changed every two to three days, following the manufacturer’s guidelines on changing media.

### 2.7. Effects of Agarose Stiffness in Spheroid Formation

Three 217-well dishes with agarose concentrations of 0.5%, 2%, and 4% (*w*/*v* in DMEM/F12) were manufactured as previously described. A549 cells were seeded on these plates at a density of 5.03 × 10^3^ cells/microwell. After 24 h, 15 microwells were randomly selected and imaged with an AmScope phase-contrast microscope. Images were then processed with ImageJ. The “free selection” tool was used to manually trace the outline of formed spheroids. To determine the number of cell clusters formed, aggregates were identified and counted. The “analyze particles” tool was used to measure cell aggregate area, and particle area for each image was summed in Microsoft Excel 2016.

### 2.8. Confocal Microscopy

Whole spheroids were fixed using 4% paraformaldehyde overnight at 4 °C and then washed with PBS three times with a five-minute incubation time each as described in Drakhilis, et al. [14] For the agarose dish, processing was done in the dish. The spheroids were then mounted on a glass-bottom MatTek dish. To clear the spheroids, an in-house prepared buffer was used based on a previously published method, Ce3D [15]. Confocal images were taken using Leica TCS SP8 confocal microscope with 40× oil immersion objective lens. In imaging the fluorescent tags, 405 and 561 nm lasers were used to detect Azurite and mCherry signals stably expressed in EAhy an HFL1 cells, respectively. Images were taken at 0.31 Airy units and a resolution of 518 × 518 pixels.

### 2.9. Multispectral Imaging Flow Cytometry

EAhy cells were seeded at a density of 1.0 × 10^3^ cells per microwell in 2% agarose and Aggrewell™800 dishes. After 24 h, the aggregates were dissociated in the dish using Accumax for 15 min at room temperature. The dissociated cells were incubated in binding buffer (140 mM NaCl, 4 mM KCl, 0.75 mM MgCl_2_, 2.5 mM CaCl_2_, and 10 mM HEPES in distilled water) with 1 µg/mL Annexin V-AF488 (Enzo, Farmingdale, NY, USA) for 15 min on ice. The samples were then washed once with the binding buffer. Then, 10 to 15 min before cytometry, the samples were stained with 1 µg/mL propidium iodide (Invitrogen, Carlsbad, CA, USA). The samples were then analyzed using the MarkII Imagestream imaging flow cytometer (Amnis, Seattle, WA, USA). RIF files were then batch processed using the IDEAS software (Amnis) and reports were created using the same software.

### 2.10. TGFβ-Induced Epithelial-to-Mesenchymal Transition

A549 cells were seeded at a density of 5.0 × 10^3^ cells per microwell in a 2% agarose dish. After 24 h, the medium was changed into basal DMEM/F12 medium supplemented with 10 ng/mL TGFβ in presence or absence of DMSO, 10 µM SB525334 (TGFβRI inhibitor, Selleckchem, Houston, TX, USA), or 20 µM Y-27632 (ROCK inhibitor, Hello Bio, Princeton, NJ, USA). After 48 h, 12 total spheroids were transferred to a gelatin-coated dish with the aforementioned treatment media. These were then allowed to migrate for 24 h and the migratory parameters were assessed via phase-contrast microscopy. Area of migration and normalized migration area were calculated based on a previously published paper [16].

### 2.11. Porcine and Murine Primary Cell Isolation and 3D Culture

All experiments were performed per the NIH Guide for the Care and Use of Laboratory Animals and were approved by Institutional Animal Care and Use Committees at Brigham Young University-Provo. A 15-month-old mouse was sacrificed and perfused intraventricularly with heparin-PBS (Greiner Bio-one, Kremsmünster, Austria). The lungs were excised, minced into fragments smaller than three millimeters, digested using STEMxyme^®^1 collagenase-dispase cocktail (Worthington Biochemical, Lakewood, NJ, USA) for one hour at 25 °C in an 800-rpm shaking incubator, and passed through a 100 µm cell strainer. The cells were then cultured using primary cell growth media. Once confluent, cells were dissociated using Accumax (Innovative Cell Technologies, San Diego, CA, USA), counted using a Bio-Rad automated cell counter, and cells were seeded at a density of 1 × 10^4^ cells per microwell into the agarose dish. The medium was changed every two to three days. The porcine lungs were gifted by Circle V Meat Co (Springville, UT, USA). Lungs were acquired within the hour of sacrifice to ensure cell viability. Cell isolation and 3D culture were then performed using the same protocol for murine lungs.

### 2.12. Image and Statistical Analyses

In a 217-well dish, microwells containing cell clusters were randomly selected. Cluster counts per microwell were determined using the ImageJ cell counter plugin. Cell clusters in each well were analyzed using the Fiji shape descriptors plugin for area, circularity, roundness, and aspect ratio [17]. Plots were generated in GraphPad Prism. Statistical significance was determined using Welch’s *t*-test within GraphPad Prism unless otherwise stated. The statistical significance marks were based on the *p*-values (ns = no significance; * *p* < 0.05; ** *p* < 0.01; *** *p* < 0.001; **** *p* < 0.0001).

## 3. Results

### 3.1. Scalable Mold Design

The ability of biocompatible hydrogels to create semi-solid molded structures from reusable silicone mold makes them uniquely suited for micropatterning applications [18]. To improve previous micropatterned hydrogel designs, three missing features were identified: (i) a minimalistic and easily scalable design; (ii) a hydrogel device that fits in standard, commercially available dishes to maximize output and minimize cost; and (iii) walls and channels to increase the efficiency of media changing to expedite 3D culture process.

The initial mold was 3D printed using the methacrylated photopolymer. This mold was then used to create a reusable silicone mold. Agarose hydrogel solution was poured over the silicone mold and allowed to harden, creating a readily available micropatterned 3D culture dish. An outline and video of the method for creating a silicone molded multi-well hydrogel plate is provided (Figure 1A and Video S1).

We designed three micropatterned plates that fit standard circular tissue culture dishes, due to the widespread use of such plates in laboratories worldwide. The device features an outer (Figure 1(Ba)) and an inner wall (Figure 1(Bc)) with a channel (Figure 1(Bb)) between the walls, which facilitates changing of media or addition of other experimental agents such as drugs, cytokines, or other media components without the loss of cell aggregates. The culture chamber is located at the center of the dish (Figure 1(Bd)) inside the inner wall. Here, cell aggregates form in microwells which have a depth and diameter of 2 mm (Figure 1(Be)). Previous research has shown that “U”-shaped microwells cause cells to aggregate into spheres, thus we used “U”-shaped well bottoms based on this research [18,19]. Using this design, three different silicone molds were created with 19, 217, and 919 microwells, each fitting into standard cell culture dishes (35, 100, and 150 mm, respectively) (Figure 1E). The design used most often in our experiments was the 217-well dish, shown as an agarose hydrogel in a standard 100 mm culture dish (Figure 1D).

During 3D culture, dissociated cells were seeded directly into the culture chamber in 3 mL of 3D growth media (Figure 1(Bd,Cd)). The maximum volume of the culture chamber is 5 milliliters and each microwell has an interior volume of approximately 8 microliters abased on our design. Due to gravity and optimal ultra-low adhesive hydrogel concentration, our design allows cells to drop into the microwells forming aggregates within 24 h. The inner wall of our design was deliberately made lower than the outer wall to aid in media changing. To change media, the user simply tilts the whole device, allowing the old media to flow over the inner wall to the channel (Figure 1(Bb)), where media is quickly aspirated without risk of losing the spheroids. The depth of the microwells prevents the spheroids from drifting out of their wells in the culture chamber when the device is tilted. Fresh media is then added dropwise onto the inner wall (Figure 1(Bc)) where it flows safely into the culture chamber with minimal disturbance to the growing spheroids (Video S2). Downstream processes like enzymatic dissociation and fixation can also be carried out in the agarose dish after draining the culture chamber of media as previously described.

To measure media change efficiency, we used 0.01 g/mL fluorescein isothiocyanate (FITC) in PBS to simulate original media that needs to be changed. The simulated medium was changed in our agarose dish and replaced with PBS equal to the original volume representing the “new media”. Samples of the fluorescent mixture were taken after every “media” solution change cycle. We compared solution/media change in our device to the 50% media change which is the suggested method for most commercially available plates including the Aggrewell™800 24-well plate (Figure 1F). Our data demonstrate that a single round of media solution change in the agarose plate shows significantly less residual fluorescence than three rounds of media change using the 50% media change method. In fact, one change of the media solution using our design is equivalent to three rounds in the Aggrewell™800 plate. Increased media change efficiency reduces the total media required for 3D culture and allows users to change media less frequently.

### 3.2. Effect of Hydrogel Stiffness in Spheroid Formation

**Figure 2:**
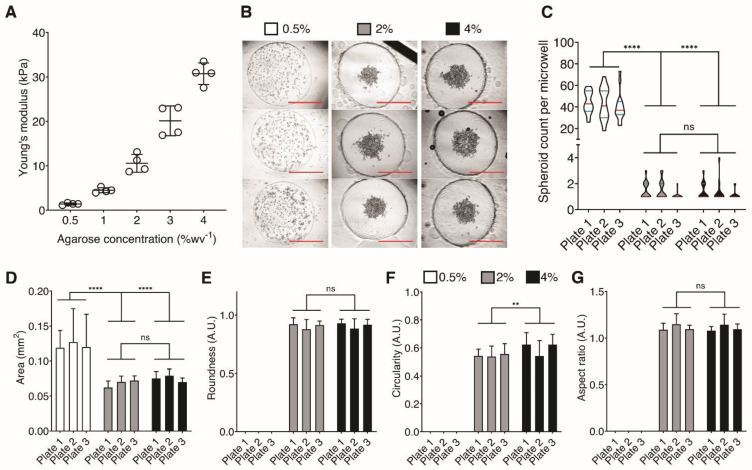
Agarose concentration and stiffness effect on spheroid formation. (**A**) Young’s modulus (kPa) estimates for each agarose concentration (n = 4 technical replicates each). Lines for each sample represent the mean +/- standard deviation. (**B**) Representative phase-contrast microscopy images of A549 tumor spheroids formed in dishes molded using 0.5%, 2.0%, and 4.0% (*w*/*v*) agarose. Cells were seeded at concentration 5.0 × 10^3^ cells per microwell and imaged 24 h post-seeding. Scale bars indicate 1 mm. (**C**–**G**) The same legend for agarose concentration found in upper middle under B is used. (**C**) Number of cell clusters formed per microwell. **** *p* < 0.001, ns = no significance, Welch’s t-test, n = 15 microwells for each agarose concentration. Red bar represents the median, with green bars the first and third quartile. (**D**–**F**) Shape descriptors for spheroid morphology, including area, aspect ratio, circularity, and roundness, respectively. ** *p* < 0.01, **** *p* < 0.0001, ns = no significant difference, two-way ANOVA and Tukey’s HSD post hoc analysis, n = 15 microwells randomly sampled from three independent plates from three independent researchers. Bars signify the mean value of each sample and error bars signify the standard deviation. (**E**–**G**) Because cells seeded in 0.5% agarose plates did not aggregate, shape descriptors for the primary aggregate could not be calculated.

Young’s moduli of agarose hydrogels with concentrations of 0.5%, 1%, 2%, 3%, and 4% were estimated through destructive compression tests using Mini Instron^®^ (Figure 2A). As expected, we found increasing hydrogel stiffness with increasing agarose concentration. We then assessed the effect of varying agarose stiffness on spheroid formation using an adenocarcinoma cell line, A549 which have been extensively used as a model for cancer and respiratory infections [11,17,20]. Twenty-four hours post-seeding, 15 randomly selected microwells were imaged using phase-contrast microscopy (Figure 2B). Then, the number of spontaneously formed spheroid aggregates in each microwell recess was quantified (Figure 2C). We observed that the number of clusters formed per well was dependent on agarose hydrogel plate stiffness (Figure 2C). There was a dramatic drop in the number of clusters formed in 0.5% (1.4 kPa) to 2% (11.5 kPa) agarose hydrogels. Additionally, there was no significant difference in cluster formation between 2% and 4% agarose dishes. To characterize the effects of agarose concentration in achieving reproducible and consistent cell aggregates, we used Fiji shape descriptors [17] to quantify spheroid formation from each agarose concentration (Figure 2D–F). The following shape descriptors were used: (1) area—since each microwell was seeded with a virtually equal number of cells, the resulting area of the cell aggregates show the extent of compaction into spheroids; (2) aspect ratio—a measure of elongation, in which a value of 1 indicates equal length on both major and minor axes; (3) circularity quantifying how much the spheroids resemble a perfect circle which has a circularity value of 1, and; (4) roundness—related to circularity disregarding local irregularities such as a jagged or smooth outline.

The total cell aggregate area per microwell was significantly lower in the 2% and 4% agarose dishes compared to that in the 0.5% agarose dish (Figure 2D). This suggests more compaction of cells in spheroids made in the 2% and 4% agarose dishes. Spherical measures including aspect ratio, circularity, and roundness were only measured for 2% and 4% agarose dishes since cells in the 0.5% agarose dish did not form a singular aggregate (Figure 2E–G). Spheroids in 2% and 4% agarose dish showed no significant difference in aspect ratio and roundness at values near 1.0, indicative of a uniform spherical structure. Circularity of spheroids grown in 4% agarose was significantly higher than in 2% agarose, which suggests smoother edges with fewer protrusions or jaggedness. Together, these demonstrate that a minimum ULA hydrogel stiffness of 11.5 kPa (2% agarose) leads to the formation of a singular, uniformly shaped spheroid in each microwell. We determined 2% agarose to be optimal in spheroid formation since it is the lowest concentration at which we observed highly significant improvements in spheroid formation and morphology. Hereafter, we performed the subsequent experiments using 2% agarose dishes.

### 3.3. Comparing Spheroid Formation in Our Agarose Dish Compared to the Aggrewell™800 Plates

**Figure 3:**
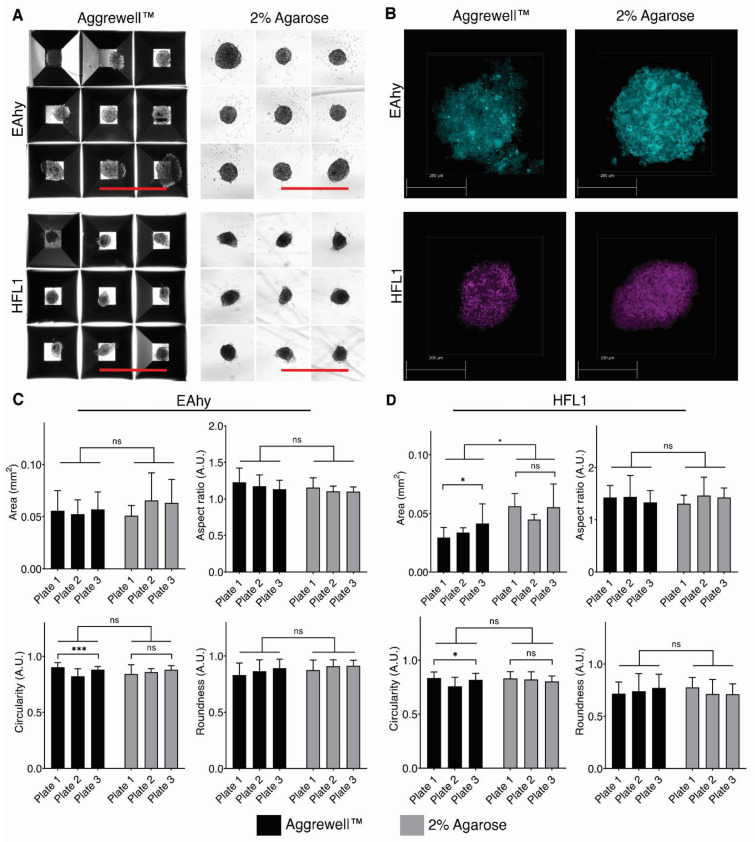
Comparison of spheroid formation in EAhy and HFL1 cells in both Aggrewell™800 and agarose dish. Each microwell was seeded with 4.0 × 10^3^ cells. The agarose dish contains 217 microwells and the Aggrewell™800 plate contains 300 microwells per well. (**A**) Representative phase-contrast images of the spheroids formed in the respective dishes. Images were taken five days after seeding. Scale bars indicate 1mm. (**B**) Representative confocal images for each cell line. (**C**) Bar plots of shape descriptors for EAhy spheroids and (**D**) for HFL1 cells. * *p* < 0.05, ns = no significance, Welch’s *t*-test of 15 randomly selected individual spheroids from three independent plates by three independent researchers. Plate-by-plate statistical significance was performed using Brown–Forsythe ANOVA, * *p* < 0.05, *** *p* < 0.001. Statistical significance was denoted only in graphs that had *p* < 0.05.

Spheroid formation in our agarose dishes was compared with commercially available Aggrewell™800 plates to assess the capability of our agarose dish as a tool for three-dimensional cell culture. Both agarose and Aggrewell™ plates were seeded separately with HFL1 cells stably expressing mCherry and EAhy cells expressing Azurite at 4.0 × 10^3^ cells per microwell. The spheroids were then cultured for five days before analysis. Phase-contrast microscopy shows Aggrewell™800 features a compact square design while our agarose dish features wider circular recesses (Figure 3A). Confocal microscopy was used for visualization of spheroid formation and characterization. (Figure 3B). We characterized the spheroids using shape descriptors from 15 randomly selected spheroids in each plate and cell type. The measurement revealed no significant differences in area, aspect ratio, circularity, and roundness for both EAhy and HFL1 spheroids between the agarose and Aggrewell™800 plates with one exception (Figure 3C). HFL1 spheroids grown in the agarose dish had a significantly larger area than those in Aggrewell™800 (Figure 3D). In summary, spheroid formation in the agarose dish is comparable to the aggregates formed using Aggrewell™800 concerning general morphology.

**Figure 4:**
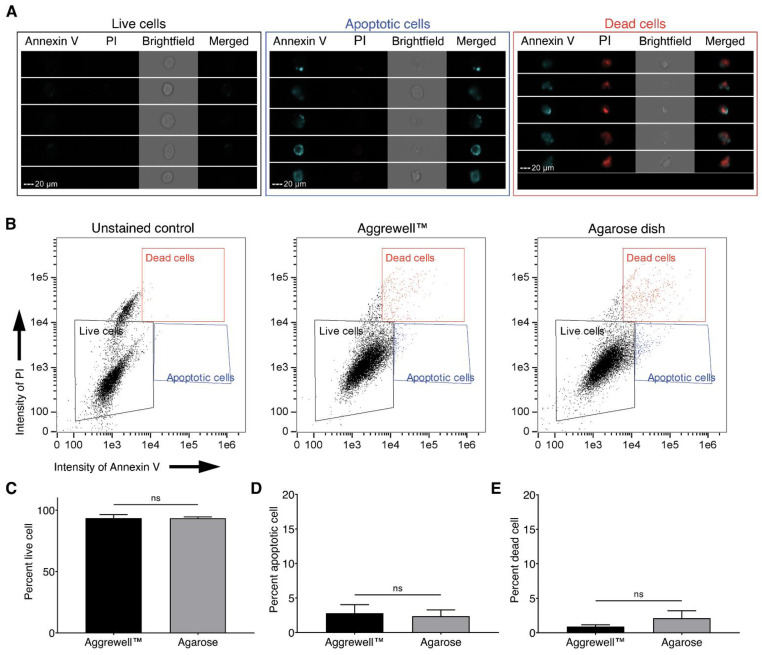
Comparing levels of cell death in Aggrewell™800 and agarose dish using imaging cytometry. (**A**) Five representative cell images from each of the gated populations. (**B**) Dot plots of dissociated cells from EAhy spheroids stained with Annexin V-AF488 and propidium iodide (PI). (**C**) Percent live cells gated (Annexin V^low^/propidium iodide^low^). (**D**) Percent apoptotic cells gated (Annexin V^high^/propidium iodide^low^). (**E**) Percent dead cells gated (Annexin V^high^/propidium iodide^high^). (**C**–**E**) Bars indicate the means and error bars indicate the SD from three independent replicates. ns = no significance, Welch’s *t*-test.

To assess differences in spheroid cell viability, we analyzed cells from spheroids cultured in our agarose dish or the Aggrewell™800 using multispectral imaging flow cytometry (MIFC) (Figure 4) [21]. Images of single cells were used to gate different populations based on Annexin V (apoptotic marker) and propidium iodide (cell viability) signals (Figure 4A). Three distinct populations were identified as Annexin V^low^/propidium iodide^low^ (live cells), Annexin V^high^/propidium iodide^low^ (apoptotic cells), and Annexin V^high^/propidium iodide^high^ (dead cells) (Figure 4B) [22]. Upon analysis, we observed that around 93% of the cells in both the Aggrewell™800 and the agarose dish were viable (Figure 4C). The difference between the percent apoptotic and dead cells between dish type were also not statistically significant (Figure 4D,E). Thus, our data suggest that the viability of cells in our agarose dish is comparable to the viability of cells in the commercially available Aggrewell™800.

### 3.4. Inhibition of EMT in the Spheroid Migration Assay

**Figure 5:**
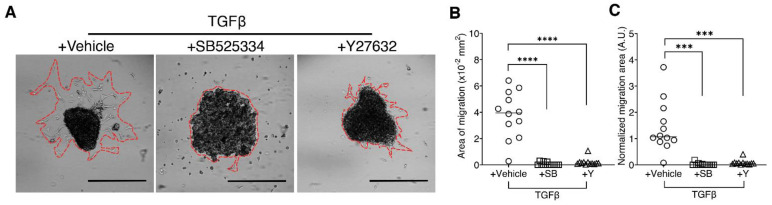
Migration assay of A549 spheroids grown and treated with 10ng/mL TGFβ in 2% agarose plates to assess the efficacy of a TGFβRI inhibitor, SB525334 (SB, 10 µM) and a ROCK-inhibitor, Y-27632 (Y, 20 µM) in inhibiting migration. (**A**) Representative phase-contrast images of treated spheroids in the migration assay. Scale bars are 250 μm. (**B**) Area of migration. (**C**) Migration area normalized to the spheroid area. (**B**,**C**) Individual points indicate individual spheroids in migration. The horizontal bar indicates the median value. **** *p* < 0.0001, *** *p* < 0.001, one-way ANOVA with Welch’s correction, n = 12 randomly selected individual spheroids. The experiment was repeated three times.

Epithelial-to-mesenchymal transition (EMT) is a major driver of many pathologies including cancer metastasis and organ fibrosis [23,24]. As such, many studies have been devoted to the inhibition of EMT. Migration assays are an accepted method in the field to measure EMT and characterize metastatic potential [25]. Previous work using biochemical, and histological endpoints showed that EMT is ameliorated with TGFβ receptor 1 (TGFβRI) inhibitor SB525334 (SB) [26] and a rho-associated protein kinase (ROCK) inhibitor Y27632 (Y) [27]. In order to show proof of concept, we used our scalable-customizable hydrogel dish along with these small molecules to inhibit the invasive properties resulting from TGFβ-induced EMT. A549 spheroids were grown in 2% agarose dishes and were treated for 48h with TGFβ plus either SB or Y. Spheroids were transferred to gelatin-coated plates and allowed to migrate for 24 h. Phase-contrast microscopy revealed the extent of migration among the treatment groups (Figure 5A). After measuring spheroid size normalized to area of migration, we observed that both SB and Y significantly decreased the migration of A549 cells on gelatin-coated plates (Figure 5B,C). Additionally, the majority of the SB-treated spheroids failed to adhere to the gelatin-coated plates. As hypothesized and shown by previous researchers, the inhibition of TGFβRI signaling by SB and the inhibition of ROCK signaling by Y was efficacious in preventing the migration of A549 carcinoma cells, even with the exogenous supplementation of TGFβ.

### 3.5. Scaffold-Free Primary-Derived Spheroid Formation

**Figure 6:**
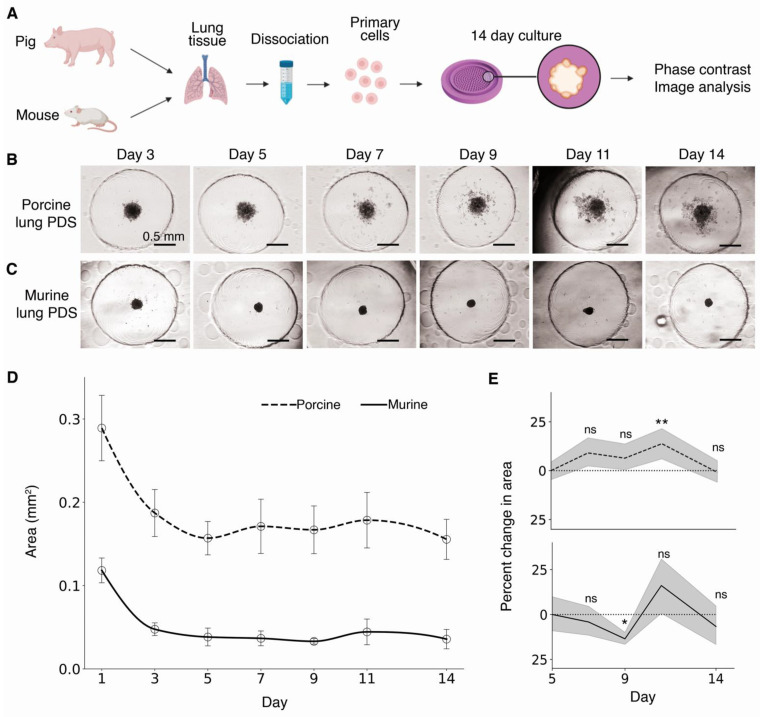
Maintenance of primary-derived spheroids in the casted hydrogel device. (**A**) Crude primary cells were dissociated and isolated from porcine and murine lungs, then grown in the device at a seeding rate of 1.0 × 10^4^ cells per spheroid. Spheroids were cultured for 14 days in the hydrogel dish. The spheroid size was monitored using phase-contrast microscopy. (**B**) Representative phase-contrast microscopy images of porcine lung primary-derived spheroids at days 3, 5, 7, 9, 11, and 14. (**C**) Murine lung PDS images. (**D**) Area of the murine-derived PDS at days 3, 5, 7, 9, 11, and 14. Dots are means and bars are 95% CI. n ≥ 25 spheroids. (**E**) Percent change in area relative to day 5 Scheme 0. * *p* < 0.05,** *p* < 0.01, ns = no significance, Welch’s *t*-test.

After validating the consistency of culture using three stable cell lines, we assessed the ability of the hydrogel dish to form and maintain primary-derived spheroids (PDSs). Somatic cell primary organoids and spheroids are part of a lucrative and exponentially expanding field in drug development and disease modeling [28,29,30]. Here, murine and porcine lung tissues were excised and digested using a collagenase-dispase buffer, grown in standard culture plates until confluence, and then seeded equally into 2% agarose micropatterned dishes (Figure 6A). The spheroid area was measured every two to three days during a 14 day culture using phase-contrast microscopy (Figure 6B,C). We observed a typical 3D aggregation–compaction pattern, with an initially larger sphere that decreases in size for a short initial period (Figure 6D) [31]. In both porcine- and murine-derived PDS, we determined day five as the end of the compaction time. Percent change in spheroid size was then normalized to day five. Size changes in both porcine- and murine-derived spheroids were not statistically significant on most days (Figure 6E). Collectively, our data show the successful use of our micropatterned device in the creation and maintenance of consistently sized and shaped spheroids from either stable or primary-derived cells.

## 4. Discussion

Using interdisciplinary expertise, we designed ULA hydrogel dishes in standard 35, 100, and 150 mm tissue culture dishes (Figure 1E). Together, the design of our agarose dish has multiple advantages over other micropatterned devices (Figure 1G). First, by using a mold to form the plates out of hydrogels allows users to modulate the ULA dish stiffness. Second, our novel design facilitates a 3-fold more efficient media exchange while leaving spheroids undisturbed. This design eliminates the need for multiple media change cycles, resulting in a reduced probability of contamination and fewer aggregate losses. Additionally, the increased efficiency and speed of media changes decrease the time and cost required to maintain 3D cultures. Third, high efficiency in media change permits fixation, enzymatic dissociation, and processing without removing spheroids from the plate. In other models, the spheroids must be extracted into vials for processing, which can result in the breakup of fragile spheroids. Fourth, the microwells in our plate design are larger with a wider visualization window when compared to Aggrewell™800 plates. The Aggrewell™800 has a visualization window of 0.0156 mm^2^ per microwell (10% of the whole microwell), while ours has the whole field of view unobstructed. Smaller imaging windows result in highly variable well brightness which impedes ease of spheroid imaging in Aggrewell™800 plates (Figure 4).

We apply the use of our micropatterned dish to form spheroids from three stable cell lines, namely A549, EAhy, and HFL1. Upon a side-by-side comparison of our dish to the Aggrewell™800, we observed little to no significant difference in the formation of a singular spheroid per microwell when using either EAhy or HFL1 cells (Figure 3 and Figure 4). Our data correlating spheroid uniformity is consistent with previous research showing that control of adhesive substrate stiffness is important in translating appropriate cellular responses (Figure 2) [31,32]. Research in fields studying the cellular response to surface tension and mechanical forces need a cell culture tool with tunability of substrate stiffness. Fixed substrate stiffness in dishes such as the Aggrewell™800 is limited in this aspect of this study.

Spheroids are an important tool in disease modeling and drug testing [33]. TGF-β inhibition is a primary target in EMT studies, especially in fibrotic diseases [34,35]. The TGF-β receptor 1 (TGFβRI) selective inhibitor, SB525334 has been shown previously to attenuate bleomycin-induced lung fibrosis [36,37]. Another recently studied drug target to combat lung fibrosis is inhibition of ROCK signaling. Our results suggest that the inhibition of ROCK signaling by Y27632 is comparable to the inhibition of TGFBRI by SB525334 in the reduction in the invasiveness of TGFβ-treated spheroids. Using our plates, we show that both SB525334 and Y27632 dramatically reduce cell migration which corresponds to invasiveness (Figure 5) [25]. Our findings support current studies on ROCK as a potential target against EMT-related diseases. For example, in A549 and MDA-MB231 cells, ROCK inhibition by fasudil significantly decreased the migration of the cells in a scratch assay [38]. Previous research on murine ROCK-haploinsufficient models shows reduced ROCK1/2 expressions protections against bleomycin-induced lung fibrosis [39]. Moreover, a separate study demonstrates the protection of mice from bleomycin injury when treated with fasudil [40]. This proof-of-concept experiment shows that our plates can be used to study the inhibition of TGFβ-induced EMT and in other drug assays.

Finally, our agarose dish is used in the formation and maintenance of spheroids derived from porcine and murine lung parenchyma. Primary-derived organoids and spheroids have shown great promise in disease modeling due to their close approximation of native organs and tissues [20]. Thus, their use in preclinical drug testing is increasing [41,42,43]. Maintaining primary-derived spheroids in culture is an important feature for hydrogel dishes, considering primary cells are often more sensitive than stable cells. We show that our molded hydrogel dish produces viable and uniform primary-derived spheroids (Figure 6).

## 5. Conclusions

We successfully created an easily scalable, minimalistic design for micropatterned hydrogel dishes that increase reliability and efficiency in 3D cell culture. The dish proved effective in studying the relationship between hydrogel stiffness and spheroid formation in A549 adenocarcinoma cells. The ability of the agarose dish to form EAhy and HFL1 spheroids was comparable to the commercially available Aggrewell™800. However, our novel design allows improved imaging and substrate stiffness modulation. As proof of concept, we performed an A549 spheroid migration assay using two investigational inhibitors against EMT. Here, we observed significantly decreased spheroid cell invasiveness upon treatment with either inhibitor. In addition to these, we maintained primary-derived cells from murine and porcine lungs for at least 14 days using the device. Together, our results demonstrate the advantages of this novel tool, which has numerous research applications using either stable or primary-derived cells to form spheroids.

## 6. Patents

Brigham Young University has filed a provisional patent for “Scalable Three-Dimensional Cell Culture Hydrogel Device.” (U.S. Provisional Pat. No. 63083791, Docket No. 2020-056).

## Data Availability

Data available upon request.

## Supplementary Materials

The following are available online at https://www.mdpi.com/article/10.3390/life11060517/s1, Video S1: Casting Plates; Video S2: Changing Media.
